# Enhancing Cognition with Video Games: A Multiple Game Training Study

**DOI:** 10.1371/journal.pone.0058546

**Published:** 2013-03-13

**Authors:** Adam C. Oei, Michael D. Patterson

**Affiliations:** Division of Psychology, Nanyang Technological University, Singapore, Singapore; University of California, Davis, United States of America

## Abstract

**Background:**

Previous evidence points to a causal link between playing action video games and enhanced cognition and perception. However, benefits of playing other video games are under-investigated. We examined whether playing non-action games also improves cognition. Hence, we compared transfer effects of an action and other non-action types that required different cognitive demands.

**Methodology/Principal Findings:**

We instructed 5 groups of non-gamer participants to play one game each on a mobile device (iPhone/iPod Touch) for one hour a day/five days a week over four weeks (20 hours). Games included action, spatial memory, match-3, hidden- object, and an agent-based life simulation. Participants performed four behavioral tasks before and after video game training to assess for transfer effects. Tasks included an attentional blink task, a spatial memory and visual search dual task, a visual filter memory task to assess for multiple object tracking and cognitive control, as well as a complex verbal span task. Action game playing eliminated attentional blink and improved cognitive control and multiple-object tracking. Match-3, spatial memory and hidden object games improved visual search performance while the latter two also improved spatial working memory. Complex verbal span improved after match-3 and action game training.

**Conclusion/Significance:**

Cognitive improvements were not limited to action game training alone and different games enhanced different aspects of cognition. We conclude that training specific cognitive abilities frequently in a video game improves performance in tasks that share common underlying demands. Overall, these results suggest that many video game-related cognitive improvements may not be due to training of general broad cognitive systems such as executive attentional control, but instead due to frequent utilization of specific cognitive processes during game play. Thus, many video game training related improvements to cognition may be attributed to near-transfer effects.

## Introduction

The link between playing action video games and enhanced cognitive and visual-perceptual abilities is well documented. However, the definition of action video games and why playing them leads to improved abilities has not been resolved.

Most action video game studies have used first person shooter games (but see [1]). These video games contain several common properties: unpredictability, intense speed, high perceptual, cognitive and motor load, the selection between multiple action plans and an emphasis on peripheral processing [2]. Action video game related perceptual enhancements have been reported in the following areas: improved peripheral vision [3], [4], [5], reduced crowding effect [6], a shorter period of backward masking [7] and improved contrast sensitivity [8]. Action video game play has also been linked to enhanced cognitive abilities such as attending to multiple objects simultaneously [3], [9], [10], [11], superior spatial skills [5] as well as reduced attentional blink effects [3]. Furthermore, these enhancements have also been seen in higher-order executive control functions such as task switching, working memory, inhibition and reduced attentional capture [12], [13], [14], [15], [16], [17].

Although there is a large amount of evidence of the benefits of action video games, not all cognitive abilities that have been measured were improved by action video game play. For instance, no differences have been found between action video game players and non-gamers in the ability to inhibit attention from returning to previously attended locations [18], a phenomenon known as the inhibition of return [19]. Furthermore, the ability to orient to an exogenous cue has generally been found to be equivalent between action and non-action video gamers [20].

Despite their potential benefits, action video games may not be suitable for everyone. Many action video games contain violent themes, making their suitability for young children questionable [21]. Hence, there is a practical need to investigate whether non-action games benefit cognitive and perceptual abilities.

Currently, little is known about the cognitive benefits of non-action video games. Two studies have demonstrated evidence of improved mental rotation skills in young children trained in Tetris [22] in addition to enhanced executive control abilities in older adults after real time strategy game training [23]. Nevertheless, these non-action video game training results are not directly comparable to studies using action video games due to differences in sample selection. While most action video game studies used young adult samples, these two studies comprised of young children [22] and elderly adults [23]. Hence, it remains unclear if young adults trained using these games will show similar cognitive improvements.

To date, there has been a paucity of studies that compared transfer effects of different genres of games within the same study. Such a comparison of multiple genres is important because it is possible that different game types will improve different aspects of cognition and perception.

In most longitudinal studies involving action video games, researchers have compared a control group playing a non-action video game (e.g., Tetris) to a treatment group playing an action video game [3], [6]. These studies have generally shown more favorable transfer effects after playing action video games compared to a control non-action video game. While the benefits of action video games are not in question, the methods that have been used in these previous studies may have favored action games. Specifically, many action video game training studies have utilized transfer tasks that mimic the demands of action video games, thus possibly maximizing transfer effects. These transfer tasks are typically fast paced, with multiple objects to track and require quick switches of attention from one target to the next. One commonly used task, the attentional blink task requires fast recovery from detecting one target (T1) in preparation for detecting a second target (T2). Cognitive abilities required in this task are highly practiced in an action video game, where failure to attend to a second target (e.g., enemies appearing in succession) may result in losing points or even the game. Similarly, the cognitive abilities required in a multiple object tracking transfer task are also frequently practiced in action video games where there are high demands to attend and respond to numerous items simultaneously to progress in the game.

Group compositions in cross-sectional studies of action video games may also have unfairly favored experienced action video game players. Specifically, in cross-sectional studies [3], [4], [10], [12], participants who reported casual playing of various genres of non-action video games have been grouped together with non-gamers in a single control group when compared with experienced action video gamers. Drawing conclusions on the superiority of action video games based on such selection criteria is problematic because the effects of playing non-action games may have been masked by the inclusion of non-gamers. Grouping game players of different genres within the same control group poses another problem. Since playing different genres of non-action video games may train different abilities, it is possible that the effects of different non-action games may cancel each other out. One way around this problem would be to directly compare performances between experienced players of specific game genres in a single study, although we suspect this would be operationally difficult because gamers tend not to play a single genre of game exclusively.

We sought to extend previous findings of cognitive improvements seen in action video games by including a wider range of video game genres. That is, in addition to an action game, we purposefully chose games that differ in cognitive demands like visual matching and search (e.g., match-3 and hidden-object game) as well as games that specifically train spatial working memory (e.g., memory matrix). We also included an agent-based life simulation game (The Sims) that did not initially appear to contain any of the aforementioned cognitive demands as a comparison.

Consistent with the claim that transfer of learning depends on the similarities between the learning and transfer task (theory of identical elements) [24], we propose that near transfer will be more likely to occur in behavioral tasks that share similar demands with the trained video games. Hence, frequent training of skills within the game itself should lead to improvements when the same skills are used in the behavioral tasks. Note that this proposal contrasts with far-transfer proposals that suggest training on one task will improve performance on multiple tasks. Far transfer claims include enhanced attentional control and speeded learning of new tasks after action video game training [25]. The near and far transfer proposals make different predictions. The former predicts that training on one cognitive task will only lead to improvements in a similar cognitive task, but the latter proposal predicts improvements in several or all tasks.

To test for these transfer effects, we included a wide range of cognitive paradigms whose demands closely mimic the demands of the video game training. This allowed us to determine the limits of how cognitive abilities can be affected by video game playing. Thus, we included measures that have previously shown improvement after action video game training (i.e., cognitive control, apprehension of multiple objects and attentional blink) as well as two tasks that contain features similar to both action and non-action video games used in the current study, but have not been tested in relation to action video game training (i.e., spatial working memory and visual search).

When a visual search and spatial working memory task were administered concurrently, performance in both declined compared to performance on each task separately [26], [27]. Hence, the inclusion of this dual task allows us to determine whether training in video games that place demands on either search skills or spatial working memory separately reduces interference between the two tasks. Finally, we included a task to assess verbal working memory span to determine if spatial working memory training can lead to far transfer effects from a visual to verbal modality.

The apparent similarity between the demands in the training games and the behavioral tasks used as pre- and post-measures allowed us to make specific predictions regarding transfer effects. First, the action game makes strong demands on flexible, rapid and multiple target detection but at the same time requires filtering out of distracting stimuli. Training should therefore transfer to attentional blink, multiple object tracking and cognitive control respectively.

In contrast to the action game where enemies are salient and require selective attention, the match-3 game demands tracking the location of multiple static items across a large area, effortful and deliberate searching for items that can be moved to make matches, and strategic planning to produce cascades of matches. To find matches requires conjunctive search of both the item color and the pattern in which it is arranged. Although at the earliest levels, the number of possible matches is plentiful so a match can be found by searching a small area of the game display, as the levels progress, the number of possible matches decreases, requiring the player to examine a larger area to find a match. There is little demand to filter out simple visual information such as color alone since matches may be made using any color at any location of the screen. Any filtering of information would be at a higher level of pattern analysis. There is a demand to keep track of multiple, separate colored groups of objects so that possible areas where matches can occur are remembered. Thus, playing match-3 should lead to improvements in visual search and the tracking of tracking of multiple static objects.

Third, the hidden-object game places demands upon visual search and thus is expected to lead to improvement in search, allowing more resources to be used for a dual task of visual search and spatial working memory. However, it does not demand fast tracking of multiple objects and thus should not improve attentional blink, cognitive control, and multiple-object tracking.

Fourth, the demands in memory matrix game are specific since only spatial working memory is trained. Although there are some memory demands in action video games to keep track of multiple items, they contain many other demands. It is thus possible that transfer will be more effective in the memory-matrix game since it has no competing demands and therefore offers the most training in spatial memory compared to other games. In addition, assuming far transfer, we predict the memory matrix game is most likely to lead to improvements in verbal working memory. This assumes that the memory matrix task trains general memory processes that are not modality specific.

Finally, for the agent-based life simulation game, we do not expect improvements in any behavioral tasks in this study since it does not appear to have similar demands to the behavioral tasks. Although it does have some memory demands to keep track of the goals of each agent, there is no incentive or need to do so as goals and objectives are available on demand in the game menu.

## Materials and Methods

### Ethics Statement

The study was approved and conducted in compliance with the guidelines set out by the Nanyang Technological University Institutional Review Board (IRB; approval code: 11/02/30).

### Participants

Participants were recruited via an online portal targeted at undergraduates at Nanyang Technological University (NTU). 75 (28 males; *M_age_* = 21.07, *SD* = 2.12) participants completed the study for course credits and S$50. Five participants dropped out during the study. Participants were randomly assigned to each training group as follows: hidden-object (n = 15), memory matrix (n = 14), match-3 (n = 14), action (n = 16) and The Sims (n = 16). All participants were not regular video game players based on self-report (defined as less than 1 hour of video game play per week in the preceding year). Written informed consent was sought from all participants prior to participation.

### Experimental Paradigms

All the experimental tasks were displayed using E-Prime 2.0 (Release candidate: 2.08.90; Psychology Software Tools, Inc, Pittsburgh, PA, www.pstnet.com). The experimental stimuli were presented to participants via a 19-inch LCD monitor from a distance of approximately 60 cm. Participants responded to experimental stimuli via a standard computer keyboard.

#### Attentional blink

**Figure 1:**
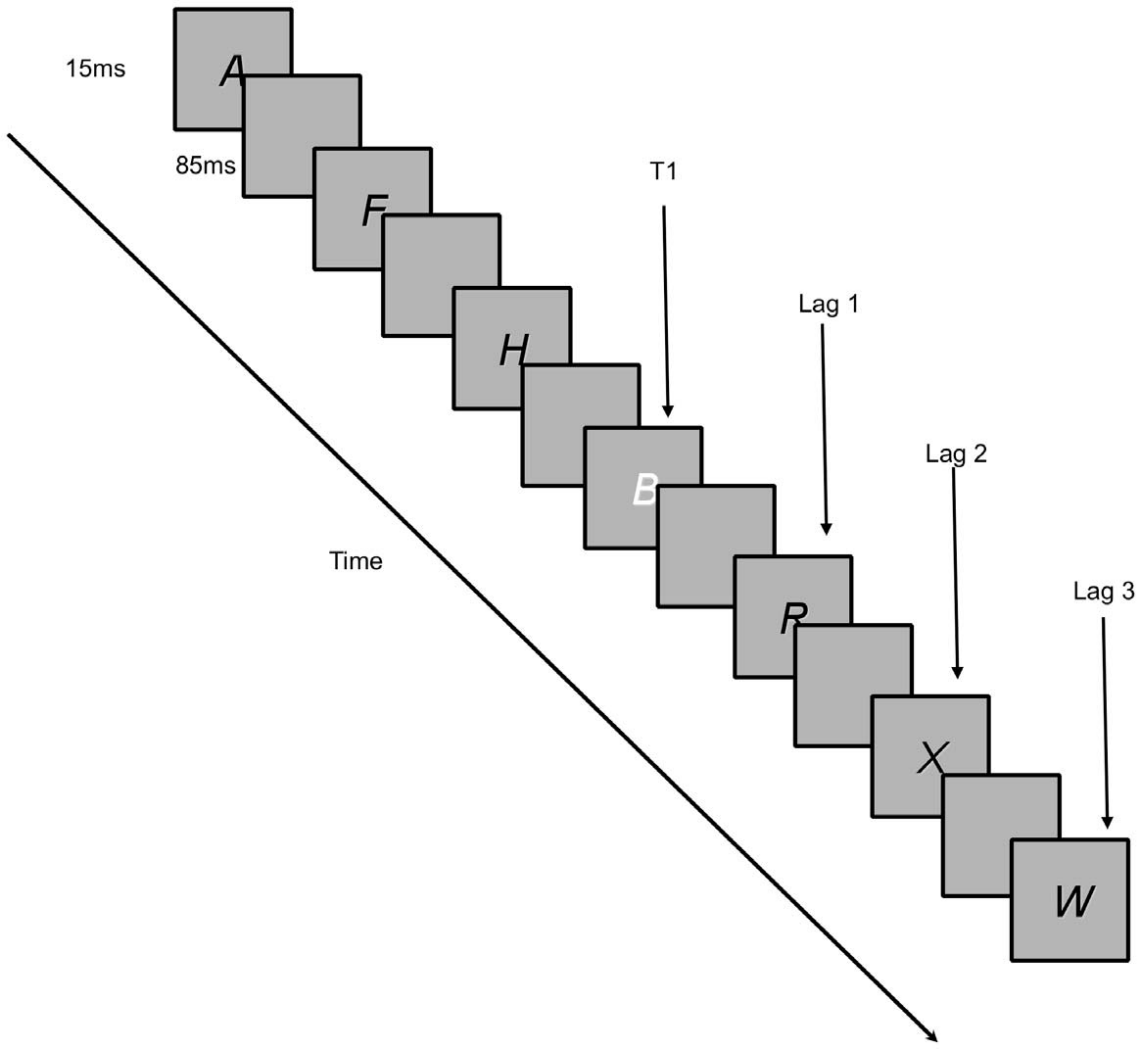
Attentional blink task. Sample of a single trial of the attentional blink task. T1 is the white letter G. In this trial, the “X” (T2) appeared in Lag 2.

As per previous studies [3], [11], the attentional blink task was used as a proxy for measuring the temporal dynamics of visual attention. In each trial, participants saw a series of black letters presented sequentially in a central location on a grey background. Between the 7th and 15th letter presented, a white letter (B, G or S) appeared. Each letter was presented for 15 ms with an 85 ms inter-stimulus interval (ISI). Participants were instructed to monitor the stream of letters presented in each trial and identify the white letter (T1). Additionally, they were instructed to decide whether a letter “X” (T2) was present in the stream of letters after T1. T2 appeared immediately after T1 (Lag 1), up to 7 letters after T1 (Lag 8) or not at all (see figure 1). T2 did not appear in 50% of the trials. Participants practiced the task for 12 trials followed by 128 test trials presented in random order. At the end of each trial, participants were instructed to indicate the target letter (T1) shown as well as whether they detected T2.

Previous studies have shown that observers often failed to detect a highly salient T2 appearing approximately 200–500 ms (Lags 2–4) after T1 was detected. This phenomenon is known as attentional blink [28].

The DV of interest here is the accuracy of detecting T2 after T1 was correctly identified. Correct T2 detections were not included when T1 was not correctly identified. In particular, we focused on Lags 2, 3 and 4 given that these lags were susceptible to the attentional blink effect.

#### Filter task

Participants were presented with an array of targets (red rectangles) and distractors (blue rectangles) in different orientations against a white background. Targets and distractors appeared in 10 different conditions of different quantities. These conditions varied from 2 to 8 targets as well as 2 to 6 distractors with the constraint of a maximum of 8 items in each array. Each array was displayed for 100 ms followed by a retention interval of 900 ms during which a fixation cross was displayed. In the subsequent display, one of the targets changed orientation in 50% of the trials. Participants responded by pressing one of two keys to indicate an orientation change or not. Participants performed 10 practice trials followed by 200 test trials.

This task was similar to that used in Ophir, Nass and Wagner [29] adapted originally from Vogel, McCollough and Machizawa [30]. The difference between the current and previous studies was that stimuli were not manipulated so that they would appear in different hemifields and thus no hemifield cue was needed.

**Figure 2:**
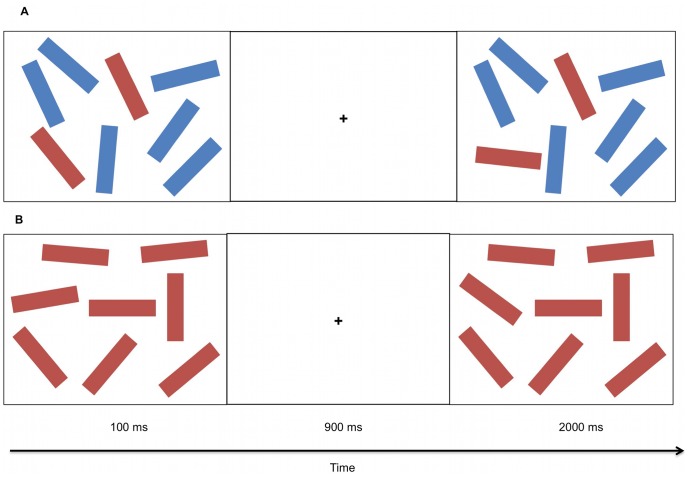
and b. Filter task. Samples from the 2 targets 6 distractors (2a) and 8 targets 0 distractors conditions (2b). Both samples include changed orientation of a single target.

We focused on two conditions in particular: the ability to filter out multiple distractors (2 target 6 distractor condition; figure 2a) and the ability to detect changes amidst a high number of targets (8 targets 0 distractor condition; figure 2b). For the 2 target 6 distractor condition, we made the assumption that due to the high number of distractors to filter out, it measured cognitive control. The ability to filter out distractors can vary between individuals [30]. Conversely, in the latter 8 target 0 distractor condition, cognitive control to filter out distractors should not have been involved due to the absence of distractors. However, owing to the high number of targets, and the requirement to allocate attention towards multiple items simultaneously, this condition demanded multiple-object tracking.

#### Visual search/spatial memory

This task was adapted from Woodman and Luck [27]. The task included three measures – a visual search task, a spatial working task as well as a dual task of visual search and spatial working memory. For the visual search task performed alone, participants saw a search array of 4, 8 or 12 squares (set sizes) each with a gap on one side of each square. On each trial, only one square was a target while the rest were distractors. Targets had a gap on either the top or bottom of the square. Distractors had gaps on the right or left sides. Participants were instructed to respond as quickly and accurately as possible with a key press when they spotted a target. Each target was mapped to a different response key. The positions of the squares depended upon the set size. For set size 4, the squares occupied the top right corner of the screen followed by four additional squares per corner in a clockwise fashion for set sizes 8 and 12 respectively. As per the original task [27], the presentation time for the array was for 4000 ms followed by a 1000 ms ISI. There were 12 trials for each set size for a total of 36 trials for the search alone condition. Participants practiced on the task for 6 trials prior to starting the experiment.

For the spatial working memory task, participants were shown a central fixation and two squares appeared sequentially, one at a time at different locations. The presentation time for each stimulus was 500 ms with an ISI (blank screen) of 500 ms. Next, a blank screen was displayed during a 5000 ms retention interval. Then a test array appeared consisting of a central fixation cross surrounded by two squares. Participants were given up to 2000 ms to indicate using a button press whether the location of the squares matched the locations of the two squares shown previously. Half the trials included squares in the matching locations. Participants were instructed to focus on accuracy instead of speed. There was a 1000 ms inter-trial interval. In the memory-alone condition, participants practiced 6 trials before 36 experimental trials.

**Figure 3:**
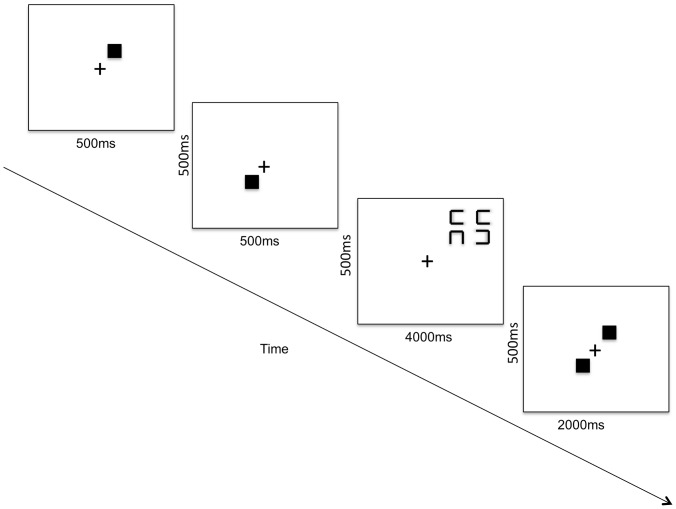
Visual search/spatial working memory task. Sample trial for dual task condition**.** Note that figure is in reverse contrast.

For the dual-task condition, participants were instructed to perform both tasks concurrently. First, two squares were displayed sequentially for 500 ms each (separated by a 500 ms ISI). During the retention interval, the participants performed the search task described above with 4, 8, or 12 squares with gaps. Next, they were shown the array with a central fixation and two squares as described above for the spatial working memory task. There were 72 trials for the dual-task condition randomly presented for each visual search set size (see figure 3).

#### Complex span

**Figure 4:**
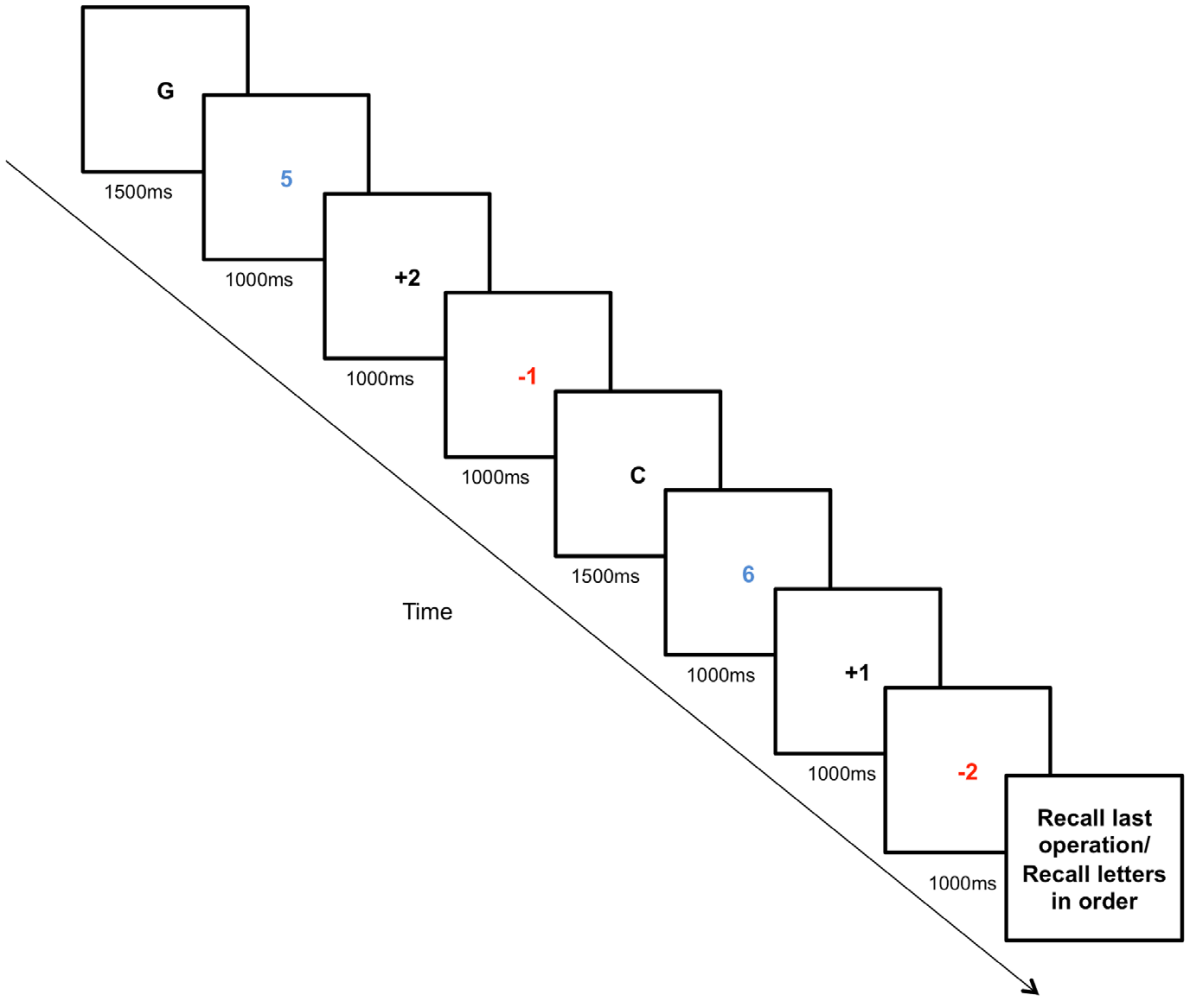
Complex span task. Sample trial for a complex span task with a two-letter load and two operations. Participants were asked to recall all the letters in order while performing the operations. In this case, one operation would be “5+2−1 = 6” and the last operation would be “6+1−2 = 5”. Hence, the correct answer in this trial is GC5.

This task is the same described in Barrouillet, Benardin and Camos [31]. Participants were requested to recall one to six sequentially presented letters while performing an arithmetic task. Every trial contained a series of letters presented sequentially and interspersed first by a base number in blue (e.g., 5) and subsequent sign-operand pairs (e.g., +2) in black. There was a minimum of one sign-operand pair and a maximum of three. The task followed a predetermined order of an increasing quantity of letters to be recalled. Within this order, the number of sign-operands pairs between each letter presented increased from one to three. A red sign-operand pair signified the end of an operation. Participants were asked to remember each letter and to compute numerical operations simultaneously. At the end of each trial, participants were instructed to enter an answer for the results of the last operation together with the letters they remembered in order (see figure 4 for an example of a trial). The total number of trials was 18 based on one to six letters to be remembered in order and one to three operations to be performed at each letter load. To limit the stress and difficulty of the operations, the sign-operant pairs were limited to either +/−1 or 2.

Although we asked participants to key in the final operation, the response was analyzed only to confirm that the participants were following directions correctly. To ensure that participants were following instructions to perform the operations, participants that did not achieve at least 70% accuracy for the final operation for either the pre or post-training task were excluded from the analyses (see results section).

One point was awarded for each letter correctly recalled in each trial. Hence, the DV was the total of all correctly recalled letters across all trials.

### Training Games

All participants utilized their personal iPhone or iPod Touch (Apple Inc) to play the games assigned to them. Interaction with the game was done using a touch-screen measured at 3.5 inches diagonally. Participants downloaded their assigned game onto their mobile devices via the iTunes App store (Apple Inc). Participants were instructed to play the games for an hour per day for 5 days each week. They were given the option to split up the hour into two half-hour playing sessions. The total duration of training was 4 weeks (20 hours in total). To keep track of their game playing, participants were instructed to input their daily playing time in an online database. Based on participants’ input, all adhered to the gameplay duration as instructed.

#### Hidden-object game (Hidden Expedition-Everest; Big Fish Games)

The objective of this game is to find hidden objects in a complex visual scene. The hidden objects are often camouflaged among other objects to reduce saliency and prevent pop-out effects. Players were instructed to touch the object on the screen when they located it. Finding objects advanced the game’s storyline.

#### Memory matrix 1.0 (Tvishi Technologies)

For this game, players were shown a 3×3 matrix in which tiles lit up in sequence. Players were then instructed to reproduce the sequence by touching the location of each tile sequentially in the matrix. Correct reproduction of the sequence increased the sequence length and matrix size. Conversely, failure to reproduce the sequence correctly resulted in decreased sequence length and matrix size. Hence, difficulty level was adjusted based on the player’s performance.

#### Match-3 (Bejewelled 2; PopCap Games)

For this game, participants were presented with different shapes (e.g, polygons, diamonds, squares, triangles and circles) of different colors in an 8×8 matrix. They were instructed to line up at least three similar colors either horizontally or diagonally by switching the positions of adjacent squares. Players earned bonus points if they achieved a cascade of matches or more than a 3-item match.

#### Action game - Modern Combat: Sandstorm (Gameloft®)

Similar to previous studies [3], [4], [10], the action video game was a first-person shooter. In this game, players controlled an in-game avatar as part of a special operations team in a war zone. The objective was to navigate in hostile enemy territory and to achieve predetermined objectives such as deactivating enemy equipment. Throughout the game, multiple enemies would appear in quick succession to engage the player. Therefore, to ensure survival in the game, players had to shoot at enemies as they appeared. Players controlled the game via virtual joysticks on screen and fired their weapons by touching a designated area on the screen.

#### Agent-based life simulation game - The sims 3 (Electronic Arts)

In this game, players controlled an in-game avatar to accomplish tasks that mimicked real-life activities. These included making friends, finding a job, sleeping, bathing, etc. The players were allowed to follow or ignore in-game objectives. Accomplishing in-game objectives required no pre-determined order.

### General Procedure

Participants performed all the tasks in a computer lab. The maximum number of participants tested in a single session was seven. Participants were first briefed about the requirements of the study. No information pertaining to their assigned training games were given during this briefing.

Following the briefing, participants performed all computerized tasks in a randomized order. The time taken to complete all experimental tasks was approximately 1.5 hours. After completing the tasks, participants were instructed to download their assigned game and play that game for the prescribed duration. After the training phase, they returned for the same set of computerized tasks. The post-training sessions were conducted in the same order with the exception of a debriefing at the end of the study.

## Results

### Attentional Blink

Since a goal of the study was to determine whether video game training can reduce attentional blink, we limited our analysis of T2 detection accuracy only to lags affected by attentional blink (lags 2–4). A 2(time: pre and post training)×3 (AB lags)×5 (training groups) ANOVA was conducted to determine if training resulted in improvements for any of these lags. There was a significant main effect of time, Wilks λ = .74, *F*(1, 69) = 23.69, *p*<.001, indicating that participants improved from pretest to posttest. This was however qualified by a training group×time interaction, Wilks λ = .80, *F*(4, 69) = 4.46, *p = *003. Interactions between lag and time, lag and training group as well as lag, time and training group were non-significant (all *p*s >.23). Similarly, between subject effects were not statistically significant, *F*(4,69) = .79, *p* = .539.

**Figure 5:**
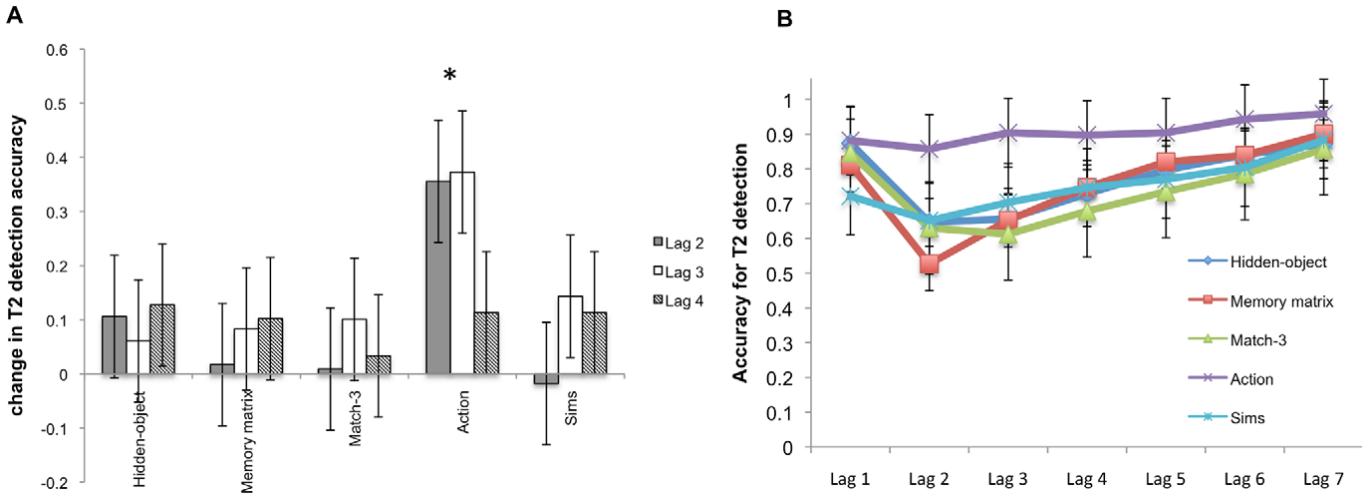
and 5b. **Attentional blink performance.** (A) Depicts changes in T2 detection accuracy from pre to post training for each training group. (B) T2 detection accuracy during post training for each training group. Asterisks represent statistically significant pre to post-training improvements. Error bars denote 95% confidence interval (CI) [51].

To determine which groups benefited from training, separate ANOVAs and follow-up paired t-tests were computed for each training group following a main effect or interaction. There was a significant main effect of time for the action game group, *F*(1, 15) = 35.80, *MSE* = .07, *p*<.001. Time×Lag interaction failed to reach statistical significance, *F*(2, 30) = .1.95, *MSE* = .02, *p* = .159. Follow-up paired t-test conducted on the action game group indicated that the improvements in accuracies for lag 2 [*t*(15) = −5.80, *p*<.001, *d* = −2.19], lag 3 [*t*(15) = −5.42, *p*<.001, *d* = −2.26], and lag 4 [*t*(15) = −3.55, *p* = .003, *d* = −1.53] were statistically significant (see figure 5a). Furthermore, unlike the other groups, the action video game group no longer showed an attentional blink since they were at equivalent levels of accuracy for all lags (all pairwise comparisons for lags 1–7 were not statistically significant, corrected for multiple comparisons; see figure 5b). Repeated measures ANOVA for all other training groups failed to achieve a significant main effect of time or time×lag interaction (all *p*s >.15).

### Multiple Object Tracking and Cognitive Control

The dependent variables (DV) in the filter task were calculated as a sensitivity index, *d’* [32] for the two conditions of interest (2 target 6 distractor for cognitive control and 8 target 0 distractor condition for multiple object tracking). Hit and false alarm rates of 0 or 1 were adjusted using the convention adopted in Stanislaw and Todorov [33].

A 2(time)×2(filter task conditions)×5(training groups) mixed ANOVA was conducted to determine if video game training improved performance in either filter task conditions. The analysis showed a main effect of time, Wilks λ = .92, *F*(1, 70) = 6.23, *p = *.015, indicating that participants’ performance changed from pre to post training. However, this was qualified by a significant time×training group interaction, Wilks λ = .88, *F*(4, 70) = 2.51, *p = *05. There was also a main effect of condition, Wilks λ = .22, *F*(1,70) = 546.243, *p*<.001 and a three-way time×condition×training group interaction, Wilks λ = .86, *F*(4, 70) = 2.80, *p = *.032. All other interactions were not statistically significant. Likewise, between subject effect were not statistically significant, *F*(4, 70) = .95, *p* = .439.

To follow-up the significant interactions, we conducted separate 2(time) ×2(condition) repeated measures ANOVA for each training group. For the hidden-object group, the main effect of time failed to achieve statistical significance, Wilks λ = .97, *F*(1, 14) = .38, *p = *.546. However, there was a main effect of condition, Wilks λ = .22, *F*(1, 14) = 51.24, *p*<.001 and a significant time×condition interaction, Wilks λ = .70, *F*(1, 14) = 5.93, *p = *.029.

For the memory-matrix group, there was a main effect of condition, Wilks λ = .11, *F*(1, 13) = 103.20, *p*<.001. However, main effect of time and interaction between time and condition failed to reach statistical significance (both *p*s >.20).

For the match-3 group, there was a significant condition main effect, Wilks λ = .20, *F*(1, 13) = 52.06, *p*<.001. But this was qualified by a time×condition interaction, Wilks λ = .70, *F*(1, 13) = 5.65, *p = *.033. Conversely, time main effect failed to achieve significance, Wilks λ = .97, *F*(1, 13) = .47, *p = *.50.

The analyses for the action game group revealed significant time, Wilks λ = .38, *F*(1, 15) = 24.58, *p*<.001 and condition, Wilks λ = .38, *F*(1, 15) = 24.63, *p*<.001, main effects with no significant interactions between the two, Wilks λ = .88, *F*(1, 15) = 2.02, *p = *.176.

Finally, for The Sims group, only condition yielded a significant main effect, Wilks λ = .21, *F*(1, 15) = 6.23, *p*<.001, with time main effect and time×condition interaction failing to reach statistical significance (both *p*s >.64).

**Figure 6:**
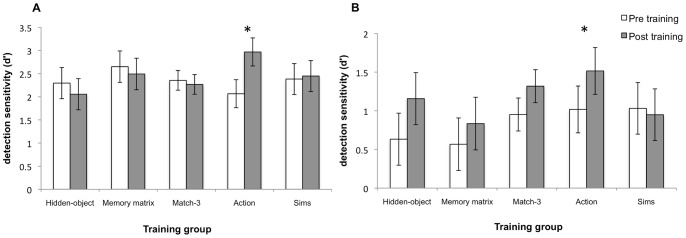
and b. Filter task performance. (A) Depicts pre and post-training performance for each training group in 2 target 6 distractor condition for cognitive control. (B) Depicts pre and post-training performance for each training group in the 8 target 0 distractor condition for multiple-object tracking. Asterisks represent statistically significant pre to post-training improvements. Error bars denote 95% CI.

Separate paired t-tests were conducted for each group that showed a significant time main effect or time×condition interactions to evaluate if performance changed from pre to post-training in the 2 target 6 distractor and 8 target 0 distractor conditions. Only the action group showed a statistically significant improvement on the 2 target 6 distractor [*t*(15) = −4.11, *p* = .001, *d* = −1.71] and the 8 target 0 distractor conditions [*t*(15) = −2.78, *p* = .014, *d* = −1.02] (see figure 6a and 6b).

### Visual Search Accuracy

For the visual search task, RT for correct detections and accuracy rates were the DVs. A 2(time) ×5(training groups) ×2(condition: single vs dual task) ×3 (set size) mixed ANOVA was conducted to determine if there were any differences in improvement in accuracy for the visual search task. Overall, there were significant main effects of time, Wilks λ = .83, *F*(1, 68) = 14.21, *p*<.001, condition, Wilks λ = .86, *F*(1, 68) = 11.23, *p* = .001 and set size, Wilks λ = .85, *F*(2, 67) = 6.176, *p* = .003. These results were qualified by significant interactions of time×condition, Wilks λ = .85, *F*(1, 68) = 11.69, *p* = .001, time×set size, Wilks λ = .91, *F*(2, 67) = 3.45, *p* = .038, as well as three way interactions of time×condition×training group, Wilks λ = .85, *F*(4, 68) = 2.92, *p* = .027. Between subject effects were not statistically significant, *F*(4, 68) = .53, *p* = .717.

**Figure 7:**
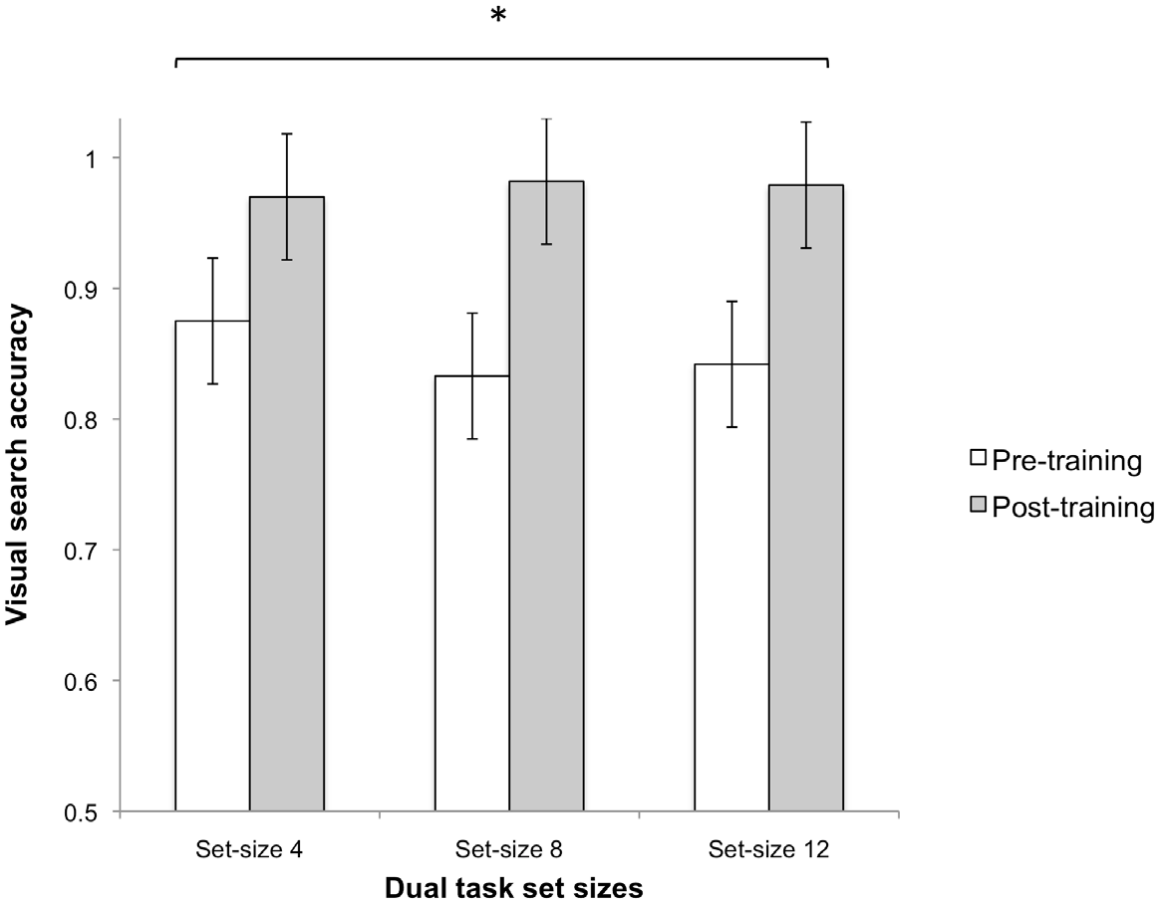
Visual search accuracy. Visual search accuracy from pre to post training for match-3 group. Asterisks represent statistically significant pre to post-training improvements. Error bars denote 95% CI.

Separate mixed-ANOVAs were analyzed for each training group to determine whether each group improved from pre to post-training. With the exception of a significant time main effect, Wilks λ = .61, *F*(1, 13) = 8.42, *p* = .012 and time×condition interaction, Wilks λ = .67, *F*(1, 13) = 6.40, *p* = .025 in the match-3 group, all main effects of time, time×condition, time×set size as well as 3-way interactions were not statistically significant for all other training groups. Follow-up paired samples t-tests for the match-3 group were conducted. The t-tests revealed that the improvement from pre to post training in the dual task condition for the match-3 training group was significant for set-size 4 [*t*(13) = −2.43, *p* = .03, *d* = −1.32], set size 8 [*t*(13) = −2.81, *p* = .015, *d* = −2.4] and set size 12 [*t*(13) = −2.80, *p* = .015, *d* = −1.35; see figure 7]. Improvements for single task conditions were not statistically significant.

### Visual Search RT

We also conducted a 2(time) ×5(training groups) ×2(condition: single vs dual task) ×3 (set size) mixed ANOVA determine if there were any differences in improvement in accuracy for the visual search task for RT. Overall, there were significant main effects of time, Wilks λ = .75, *F*(1, 68) = 22.94, *p*<.001, condition, Wilks λ = .32, *F*(1, 68) = 141.63, *p*<.001 and set size, Wilks λ = .05, *F*(2, 67) = 703.39, *p*<.001. Between subject effects were not statistically significant, *F*(4, 68) = .76, *p* = .557. These results were qualified by significant interactions of time×condition, Wilks λ = .94, *F*(1, 68) = 4.70, *p* = .034, and condition×set size, Wilks λ = .84, *F*(2, 67) = 6.49, *p* = .003. All other interactions failed to reach statistical significance. Separate ANOVAs were analyzed for each training group to determine whether there were any pre-post training reductions in RT. For the hidden-object group, there were significant main effects of time, Wilks λ = .36, *F*(1, 14) = 24.41, *p*<.001 as well as an interaction between time and condition, Wilks λ = .45, *F*(1, 14) = 17.33, *p = *.001. Significant time main effects were also seen in the memory matrix group, Wilks λ = .36, *F*(1, 13) = 22.97, *p*<.001, but there were no significant interactions. There was also a significant main effect of time for the match-3 group, Wilks λ = .53, *F*(1, 13) = 11.70, *p* = .005 as well as significant time×condition×set size interaction, Wilks λ = .41, *F*(2, 12) = 8.73, *p* = .005. All other main effects or interactions with time were not significant.

Paired t-tests were conducted on the hidden-object, memory matrix, and match-3 groups to examine pre to post training improvements in search time. The hidden-object game group had a significant reduction in RT for all set sizes in both single and dual task conditions [search-only condition of set size 4, *t*(14) = 3.95, *p = *.001, *d = *1.54, set size 8, *t*(14) = 2.50, *p* = .025, *d* = 0.91 and set size 12, *t(*14) = 2.33, *p* = .035, *d* = 0.85; dual-task condition of set size 4, *t*(14) = 6.38, *p*<.001, *d* = 2.49; set size 8, *t*(14) = 4.92, *p* = .001, *d* = 1.81; set size 12, *t*(14) = 4.40, *p = *.001, *d* = 1.91].

The memory matrix training group had a reduction in RT in set size 4 [*t*(13) = 2.38, *p* = .034, *d* = 1.23] and set size 8 [*t*(13) = 3.19, *p* = .007, *d* = 1.21], for the search-only conditions. There was a significant reduction in RT for all set sizes in the dual task condition [set size 4, *t*(13) = 2.78, *p* = .016, *d* = 1.16; set size 8, *t*(13) = 4.00, *p* = .002, *d* = 1.67 and set size 12, *t*(13) = 2.64, *p* = .02, *d* = 1.09].

**Figure 8.Visual:**
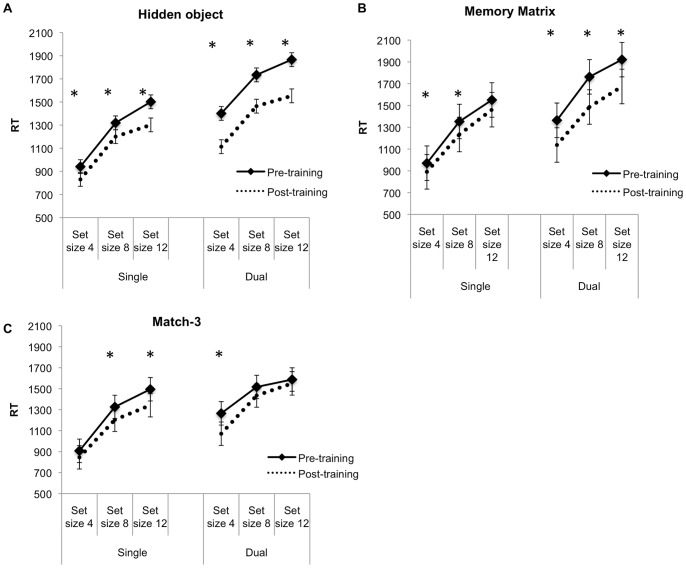
Visual search RT. Pre and post training search RT for hidden-object (A), memory matrix (B) and match-3 (C) groups. Asterisks represent statistically significant pre to post-training improvements. Error bars denote 95% CI.

For the match-3 training group, there were significant RT improvements seen in search-only set size 8 [*t*(13) = 3.16, *p* = .008, *d* = 1.21], and set size 12 [*t*(13) = 2.71, *p* = .018, *d* = 1.02]. For the dual-task condition, there was a reduction in RT for set size 4 [*t*(13) = 3.04, *p* = .009, *d* = 1.34] (see figure 8).

### Spatial Memory Task Accuracy

For the sake of simplicity, rather than include task condition (single vs dual task), we coded the memory alone condition as set-size 0 in our analysis, thus eliminating one factor. The resulting analysis was a mixed 2(time) ×5(training groups) ×4 (set sizes 0 = memory alone; 1–3 = dual task conditions) ANOVA. The results of the ANOVA indicated a significant main effect of time, Wilks λ = .75, *F*(1,69) = 23.27, *p*<.001. There was also a main effect of set-size, Wilks λ = .39, *F*(3,67) = 35.20, *p*<.001. These main effects were qualified by a significant time×set size interaction, Wilks λ = .78, *F*(3,67) = 6.38, *p* = .001. All other interactions failed to achieve statistical significance (all *p*s = >.30). The between-subject training group effect was also not statistically significant, *F*(1,4) = .69, *p* = .601.

**Figure 9:**
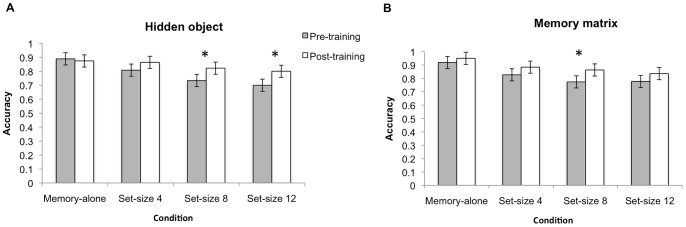
Spatial working memory accuracy. Spatial working memory performance from pre to post training for hidden-object (A) and memory matrix (B) groups. Asterisk represent statistically significant pre to post-training improvements. Error bars denote 95% CI.

Separate 2(time) ×4(Set size) ANOVAs were analyzed for each training group to determine whether there were any pre-post training improvements in accuracy for the spatial memory task. For the hidden-object group, there was a significant time main effect, Wilks λ = .72, *F*(1,14) = 5.34, *p* = .037 as well as a significant time×set size interaction, Wilks λ = .33, *F*(3,12) = 8.11, *p* = .003. Separate paired t-tests indicated that the hidden-object group improved in the dual-task condition, set sizes 8 [*t*(14) = −3.13, *p = *.007, *d* = −1.19] as well as set size 12 [*t*(14) = −3.29, *p* = .005, *d* = −1.21] (see figure 9a).

For the memory matrix training group, there was a significant main effect of time, Wilks λ = .64, *F*(1,13) = 7.22, *p* = .019 but a non-significant time×set size interaction, Wilks λ = .66, *F*(3, 11) = 1.93, *p* = .183. Planned paired t-tests indicated that only dual-task spatial memory accuracy set-size 8 had a significant improvement [*t*(13) = −2.28, *p* = .04, *d* = -.90] while dual task set size 12 was marginally non-significant [*t*(13) = −1.91, *p* = .078, *d* = .79] (see figure 9b). All other main effects and interactions for other training groups not mentioned here failed to achieve statistical significance.

### Complex Span

To ensure that the participants gave adequate attention to both arithmetic and verbal memory tasks, we limited the analysis to only participants that achieved 70% accuracy for the final operations. In all, we excluded 20 participants from subsequent analyses who did not make this cut-off. Four participants each from the hidden-object, match-3 and action game groups were excluded while three and five participants were excluded from the memory matrix and The Sims groups respectively. The average percent correct for the last operations for each of the remaining groups for pre and post-training was at least 85%.

As each letter load consists of one to three operations, a repeated-measures ANOVA at each level of the time factor compared the scores of the three operations to determine if performance differed. The results indicated that performance did not differ across the three operations for pre-training, *F*(2, 108) = .17, *MSE = *14.33, *p* = .85 and post-training, *F*(2, 108) = .15, *MSE = *13.89, *p* = .86. As a result, we combined the scores obtained from each of the operations for each letter load in our subsequent analyses.

The dependent variable is the total score calculated from all 18 trials. We first conducted a 2(time) ×5(training group) mixed ANOVA to test for performance changes and if changes differed between groups. The analysis showed a significant time main effect, Wilks λ = 81, *F*(1, 50) = 11.94, *p* = .001. However, this was qualified by a time×training group interaction, Wilks λ = .75, *F*(4, 50) = 4.21, *p* = .005. Between subject effects were not significant, *F*(4, 50) = 1.08, *p* = .376.

**Figure 10:**
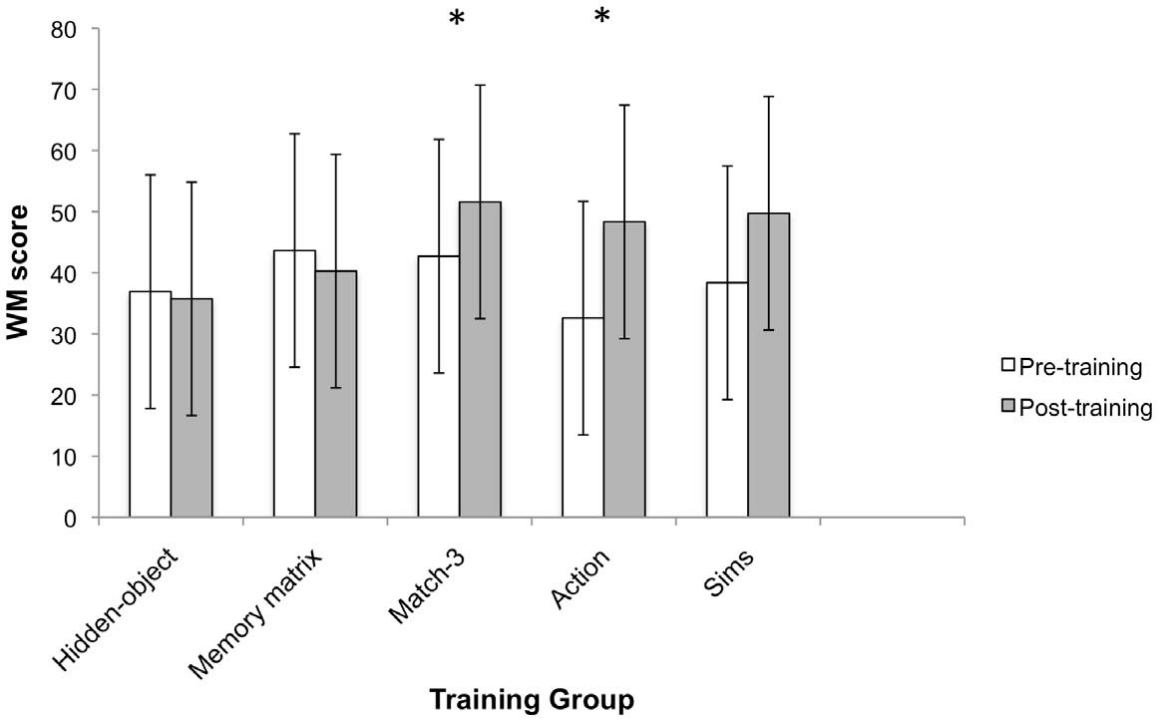
Complex verbal span performance. Complex verbal span performance from pre to post training. Asterisks represent statistically significant pre to post training improvements (α ≤.01). Error bars denote 95% CI. Note that The Sims group was marginally non-significant after Bonferroni correction of α –level.

To follow up the significant interaction, we conducted paired *t*-tests to determine if each training group improved performance from pre to post-training. However, α-level for statistical significance was adjusted using Bonferroni corrections for the number of comparisons made (.05/5 = .01). The paired *t*-tests revealed that the match-3 [*t*(9) = -3.36, *p* = .008, *d* = −1.51] and action groups [*t*(11) = −3.24, *p* = .008, *d* = −1.78] improved significantly, while the improvement for The Sims group [*t*(10) = -2.63, *p* = .025, *d* = −1.13] was marginally non-significant after the Bonferroni adjustments (see figure 10). All other paired *t-*test revealed no significant improvements in span performance for the memory matrix group and the hidden-object groups (all *p*s ≥.74).

Note that we also analyzed the results without excluding the 20 participants that did not make the cut-off because they did not achieve the 70% criterion for the arithmetic operations. The significant results when including the 20 participants were equivalent.

## Discussion

One aim of this study was to determine whether playing non-action video games enhanced cognitive performance. We confirmed that cognitive improvements following video game playing were not limited to action video games alone. The hypothesis that transfer would take place when game demands closely matched behavioral task demands was also confirmed. Additionally, we demonstrated that video game-related enhancements to cognition occurred despite using mobile devices for training, which had much smaller screen sizes than desktop or laptop computer screens. We will discuss performance on each task separately before examining the improvements from a larger vista.

### Attentional Blink

Only the action game group showed post-training improvement in detecting T2 during the period normally affected by attentional blink. These results supported the hypothesis that transfer is likely when the game and task share common elements or demands, and corroborated previous work showing that action video game training can improve recovery from attentional blink [3], [11]. In fact, playing the action game appeared to eliminate the attentional blink effect altogether, at least within the limits of how it was measured in the current study The complete elimination of the attentional blink is surprising since the training regime used in this study was not better controlled nor was it a more intensive training regimen than previous studies. On the contrary, compared to previous studies [3], [6] the current study was designed to allow more freedom to participants to play games in their own time at the location of their choosing. Hence, it is unclear whether complete elimination of attentional blink could be expected in future studies using an identical paradigm. Nevertheless, the results of action game training-related reductions in attentional blink are consistent with previous investigations [3], [11].

How might playing an action video game improve attentional capacity so that temporally close targets can be detected without a reduction in accuracy? One possible mechanism is an improvement in the speed and control of switching attention between targets [34], [35]. Note that the need to switch attention rapidly amongst targets is a highly practiced skill in an action game because the player must frequently respond to enemies that appear in quick succession. In contrast, the other training games do not demand such rapid switches of attention. Thus, this frequent practice and the increasing demand to switch between temporally close stimuli as the action video game progresses may have resulted in a reduction of the attentional blink effect via an improvement in attention switching. This suggestion is further supported by results showing that experienced action video gamers and those trained in action video games were better able to rapidly switch between stimuli compared to controls [16], [17].

### Multiple-object Tracking

Matching the near transfer hypothesis of common elements between the game and task, the action game group improved in multiple-object tracking. This finding is consistent with previous work that showed regular action game players and those trained using action games apprehended more multiple objects simultaneously compared to non-gamers or those trained using non-action video games [3], [9], [10], [11].

The training regime in the current study used a much smaller touch-screen as a display compared to previous studies. The fact that the behavioral task used a screen a magnitude larger than the training indicates that the training improved performance in parts of the visual field that had not been explicitly trained. This finding corroborates earlier work that showed that useful field of view was improved by action gaming that extended to the regions in the periphery that were not trained in typical action game play [3].

What are some possible mechanisms for improvements in multiple-object tracking? In the action video game used for training in this study, players had to keep track of and respond quickly to multiple objects in their focus of attention (e.g., multiple enemies, goals, paths, items to be retrieved). Hence, as a result of regular action game play, it is plausible that the participants became more efficient in their ability to track multiple fast moving objects in parallel, an ability that transferred to tracking static objects in the transfer task.

In contrast, there was no transfer from the match-3 game players possibly because match-3 differed from the action game in two important ways. First, for match-3, the number of objects to track did not progressively increase. There were always 64 objects on view. Although there was an increasing speed pressure as the game progressed, the number of items to track remained constant. Second, the objects only changed position due to the player’s actions, and were predictable and mostly static. Thus, the lack of transfer compared to the action video game may be due to the fact that only tracking moving objects was demanding enough to lead to increased capacity, or that there was no progressive increase allowing players to master tracking at an easier level before moving on to a more difficult level. Further experiments are necessary to decide between these possibilities.

The memory matrix group also did not show any improvement in keeping track of multiple objects. This is somewhat surprising since on the surface, memory matrix should match well with the multiple-object tracking task. The number of items to remember is progressively increased as the participant improves in the memory game. However, the training was different from the behavioral task in that a) it required memory for a sequence of items each individually, rather than the necessity to keep track of multiple objects simultaneously; b) it is repetitive without any changes to the presentation and context of the stimuli. Hence it is possible that the repetitiveness of the game led to specificity in learning that made improvement only possible within the game itself or an extremely similar task to the game [36]. In addition, the participants may have developed strategies that work for encoding each item individually, but not for encoding multiple items quickly as demanded by the behavioral task. These explanations are speculative and will require further examination.

### Cognitive Control

Our results indicate that only training by playing an action video game improved cognitive control for filtering out task-irrelevant stimuli. This corroborated previous finding of improvements in visual selective attention as a result of action video game play using different experimental paradigms to measure cognitive control [3], [12], [13].

Recent research suggests probable mechanisms for the superior performance in visual selective attention as a result of action game play. It has been shown that action video gamers were less prone to distraction by task-irrelevant stimuli than non-gamers [12]. Two possible explanations were proposed for these results. One is that action video game players have enhanced top-down control and thus they were better able to filter out distractors. Alternatively, action video gamers could be better able to recover from attentional capture compared to their non-gaming counterparts [12]. A recent study supported the former assertion that action video game players were less susceptible to attentional capture. Specifically, using an eye-tracker, it has been demonstrated that regular action video game players had significantly lower proportions of initial saccades to an abrupt onset stimuli compared to their non-action gaming counterparts [13].

A recent electrophysiological study provided evidence for the neural mechanisms for the superiority of top-down control in action video gamers. Using steady-state evoked potentials (SSVEPs), action video gamers showed increased suppression of SSVEP amplitudes to unattended peripheral stimuli compared to non-gamers. Furthermore, suppression was associated with faster speed and greater accuracy for target detection in the action gamers. Additionally, action gamers showed larger amplitude of the P300 component in event-related potentials (ERP) compared to non-gamers for attended targets [37]. The P300 amplitude is related to detection of a stimulus and the decision process related to it [38]. These findings coupled with greater suppression of SSVEP amplitudes to peripheral non-targets therefore indicated that not only were action video game players better able to suppress distractors, they were also better at target detection. The fact that action game players in the current study improved both the abilities to detect multiple targets and filter out irrelevant targets corroborated this study.

Additional evidence of neural mechanisms showing greater attentional control for filtering of distractors in action gamers was also recently demonstrated using fMRI. In a visual search task, moving distractors led to less BOLD activity in motion sensitive regions (MT/MST) relative to non-action gamers [39]. This suggested that action video gamers were more efficient at suppressing the processing of task-irrelevant stimuli. Additionally, in non-gamers, frontal-parietal areas, which included the superior frontal sulcus as well as intraparietal sulcus and dorsal anterior cingulate gyrus showed increased activations when attentional demands increased whereas in action gamers, these regions were minimally activated. These results implied that action gamers might have automated the allocation of top-down attention so that it required much less effort than non-action video game players [39]. Taken together, these investigations indicated a causal relationship between playing action video games and the ability to suppress task-irrelevant distractors.

Although improvements in top-down directed distractor suppression after playing action games appeared to be a far transfer effect since on the surface the task and game did not resemble each other, we argue that the transfer occurred because there were common underlying demands between the game and the behavioral task. Specifically, the ability to suppress task-unrelated distractors is crucial in a stimuli-rich visual environment within an action video game. To perform successfully within the game, the player must apply top-down control to filter out large amounts of irrelevant stimuli and focus on multiple targets on the screen. Failure to filter out distractors may result in an inability to progress within the game. Hence, the common elements hypothesis suggesting a limited transfer effect remains valid to explain the action game related transfer effects on cognitive control, specifically on the ability to inhibit distractors. We suggest that these common elements are at a cognitive stage of processing rather than a perceptual or motor stage. Thus, it is expected that the enhancements in performance will transfer to many tasks that vary widely on perceptual and response demands as long as they retain the same underlying cognitive demands.

### Visual Search and Spatial Working Memory

Previous research showed that performing a visual search task concurrently with a spatial working memory task adversely impacted performance in both tasks compared to performing each task separately [26], [27]. We replicated these findings by showing reduced accuracy for the spatial working memory task and increased RT for visual search as participants performed the dual task compared to performing each task separately. This may reflect dual-task interference within a limited-capacity mechanism such as visuospatial attention [40], visual working memory [41], [42] or a system representing spatial locations [27] that is shared between search and detection of location change in the spatial working memory task.

In the current study, hidden-object and memory matrix training significantly improved both visual search and spatial working memory while match-3 training improved visual search, supporting our earlier predictions. For the hidden-object game training, although we did not find a significant improvement in search accuracy in any of the set-sizes, search performance was enhanced as shown by significant search time improvement. As accuracy was very high (at least 90%) in all but the dual task set size 12 condition, it is plausible that a ceiling effect limited the possibility of finding of an improvement.

The search time improvement in the hidden-object game group was unexpected because the game did not require speeded search. Yet, training resulted in improved search efficiency. It is important to note that the search for targets in the hidden-object game was arguably more difficult than the visual search behavioral task as the items-to-be-found in the game were not predictable and the visual scene was far more complex and varied. Furthermore, the hidden objects were often camouflaged within the colored background unlike the visual search task. Hence, frequent search for varied and novel targets in the hidden-object game may have led to improved efficiency in top-down biasing of search [43].

Training in the hidden-object game led to improved spatial working memory accuracy. Crucially, the improvements were seen only in the dual-task conditions, although the lack of improvements in the single task condition may have been due to a ceiling effect. Nevertheless, taken together with improvements in search efficiency, these results suggest that the hidden-object training led to improved search so that additional cognitive resources in the aforementioned hypothesized limited-capacity mechanism were available to perform the spatial working memory task.

As expected, the memory matrix group also showed improvements in the spatial working memory task. Specifically, while memory matrix training did not improve accuracy rate for every condition in the spatial working memory task, there were accuracy improvements for the dual-task set size 8 condition. Additionally, for the visual search task, the memory matrix group decreased search times for search-only set-sizes 4 and 8. Importantly, there were also significant reductions in RT for *all* set-sizes in the dual task condition when both visual search and spatial working memory were required.

How would memory matrix training improve spatial working memory and visual search? One fundamental difference between the memory matrix game and the other games in this study is the heavy reliance of spatial working memory to successfully play the game. Hence, assuming that the shared capacity-limited resource is visual working memory as suggested [41], [42], memory matrix training may have enhanced visual-spatial working memory so that both location change detection and visual search performance were improved. This game was unique in the sense that visual-spatial working memory was the only skill that was explicitly trained in the game itself, unlike the other games that had multiple demands.

As expected, the match-3 game training group also showed improvements in visual search accuracy and search time as a result of training. These improvements in accuracy were seen in all set sizes in the dual-task condition while search time decreases were seen in search-only set size 8 and 12 as well as dual-task set size 4 conditions. We hypothesized that improvements in search and visual working memory would occur due to characteristics of the match-3 game, Bejeweled 2. Within the game, players were expected to search the entire visual scene to locate jewels that matched in color and in shape. Hence, it is likely that general visual search skills were also actively engaged and trained during playing. Another important factor that might contribute to learning and transfer in the game is the increasing difficulty in searching within the game. As a player advanced to higher levels, the number of possible matches within the 8×8 matrix decreased which led to a lower chance of finding a match within any one area of the matrix. This gradual increase in search difficulty is important as learning and transfer is more likely when the task is optimally challenging to the learner [44]. Thus, other variants of match-3 (e.g., Bejewled Blitz) that do not change in difficulty, may not lead to improvements in visual search.

### Complex Span

The complex span task was administered to determine whether training in any of these games would transfer to complex verbal working memory tasks [45]. We predicted that memory matrix, the only task that focused on memory alone, would be most likely to transfer if the improvement was for a modality-free process of memory. Contrary to this hypothesis, no significant improvements were seen in the memory matrix group. Unexpectedly, improvements in the complex span task occurred in the match-3 and action video game training groups.

The improvements by the match-3 and action game groups indicated that training in these two games might lead to improvements in higher-order executive processes which are crucial for performing complex span tasks. Unlike simple working memory span tasks that emphasize only storage and retrieval, complex span tasks are dual-tasks that recruit additional cognitive processes, such as executive control of attention to allow for efficient switching between tasks and to increase focus on current tasks in the presence of distractors [46], [47]. Previous neuroimaging research indicated that performance in complex span tasks such as the one used here actively recruited the left dorsolateral prefrontal cortex, an area linked with executive processes, but not when either the span or arithmetic tasks were performed in isolation [48]. Hence, these two games may have both frequently recruited and trained higher-order executive function, including task switching and strategic planning. First, for the action game, players switched between concurrent tasks like engaging sudden onset enemies while accomplishing several mini-objectives within the game, which in turn required holding the objectives in working memory. Similarly, performance on the match-3 game also required keeping track of matching items in working memory while formulating movement plans. These plans may occur several moves in advance. Hence, training using these games may have led to improvements in executive processing, which translated to improvements in the cognitively demanding complex span task. Further investigations are necessary to determine on the transferability of executive improvement from training to novel tasks.

In contrast to the match-3 and action video game training, we argue that memory matrix training required neither strategizing, nor anticipatory planning. In memory matrix, there is always only one fixed order of presentation. Furthermore, memory matrix training appears more similar to a simple span, rather than a complex span task. This may explain why no improvements were found for the memory matrix group in the complex span task. Unexpectedly, in the agent-based simulation group, post-test improvement in complex span trended towards significance. While the results are tentative, we speculate that playing this game may also have led to improvement in executive processes, since the game included planning and strategizing elements. Players were required to hold active representations of future plans in working memory while performing other tasks. Hence, playing The Sims could have trained executive functions like the action video game and match-3, albeit to a lesser extent since there were fewer time demands. It is plausible therefore that a longer and more intense training regimen may be necessary to achieve similar results to the faster-paced games. Interestingly, a recent study supported this possibility by reporting that 50 hours training in the game The Sims improved the task-switching aspect of executive functioning [16].

### Limitations

There were a number of limitations in the current study. First, unlike previous investigations that required participants to perform the training tasks in a laboratory [3], [6], participants in the current study were allowed to train on the games at their own leisure. Although participants entered daily logs of their gameplay, it was not possible to ensure actual fidelity to the prescribed training regimen. However, allowing participants to train during their own free time had the advantage of being more applicable to how the games were played normally. In addition, the results using action games replicated results of lab-controlled studies.

Second, some of the games may have drawn more interest from the players than others leading to non-specific factors such as motivational differences rather than the properties of the game accounting for behavioral change. It is known that action video games are highly motivating and arousing [49], [50]. In contrast, games like memory matrix were repetitive. However, since different groups showed improvements in different tasks, it is unlikely motivation and arousal differences could have explained the cognitive improvements. Additionally, even games that were repetitive showed transfer to cognitive tasks, confirming that strong interest and variability in gameplay alone were not vital for transfer to occur.

### Conclusions and Future Directions

The results from the current study corroborated the increasing body of evidence indicating that action video game play enhances several cognitive and perceptual abilities. In contrast to other studies using larger screen sizes, the results here showed that these aspects of attention and verbal span were similarly improved with a small screen size on a handheld device. In addition, rather than training in a lab, participants self-controlled play time. This has implications outside the laboratory with the increasing popularity and market penetration of smart phones and handheld digital devices.

Novel findings were also reported by including several video game training groups. Although previous studies had found only limited evidence of transfer after playing non-action video games, most studies included transfer tasks that closely mimicked the demands in action video games, favoring transfer for these games. In the current study, one example was the demand to detect rapidly appearing targets that was shared by both the action video game and the attentional blink task. Thus, the success of action video games at transferring to many tasks may have been due to the common perceptual and cognitive skills shared between action video games and transfer tasks. To further test this hypothesis requires using a variety of action video games that contain different combinations of demands. The inclusion of multiple games with varying demands demonstrated that when a wider battery of transfer tasks was included that contained demands closely matched to the training games, transfer effects favoring other game types could be achieved as well. Thus, we suggest that transfer effects are maximized when the transfer task contains elements that are similar to the training game [24].

However, although a theory of identical elements [24] can explain the transfer effects in this study, a major gap remains. The theory does not specify at what level the identical elements must occur in order for transfer to be successful. One possibility is the requirement that the training and transfer tasks share similar cognitive and/or perceptual demands. For instance, the hidden object game and visual search tasks share similar perceptual demands to search the visual scene. Another approach to explain transfer is that the more general the trained process (as opposed to a process that is specialized for use in only one task), the greater number of tasks that will show improvement. An example of a general process is working memory, something that may be recruited for almost any complex cognitive behavior. Other general processes include early sensory processing, so that almost any task involved in that modality of sensory processing could be improved. Improvement in visual acuity is an example of a general early sensory process improvement.

Overall, our results were consistent with many previous studies that showed that action video games led to the most varied transfer. Hence, it is also plausible that action-video games trained overall high level general processes like attention or learning mechanisms, and this general level of learning then transferred to allow improvement each task that recruited these general processes [25]. Future work should be aimed at resolving or reconciling these competing theories of transfer. These findings may in turn have implications to allow better design of training tasks to maximize transfer.

A key takeaway from the results here is that playing non-action games can lead to transfer. As action games tend to be of the violent, fast-paced, first person shooting genre, not everyone may be inclined to play. Much work remains to be done to specify what kinds of cognitive processes can be trained by non-action video games. It is important to increase the battery of games that can be used to train cognition and perceptual skills, both to increase the number of cognitive functions that can be improved, and also to allow participants to choose their preferred style of game.

In closing, our results here, taken with previous cross-sectional and longitudinal studies strongly suggest a causal effect of video game playing on human cognitive and perceptual enhancement. Additionally, these findings indicated that different game genres have positive effects on different cognitive skills. This has clear practical benefits because it suggests that different video games can be selected depending on which cognitive skill one aims to improve. This may have applications in enhancing specific aspects of cognition in areas such as cognitive rehabilitation and occupational training. Currently, to our knowledge, it is not known whether there is a limit to the number of different skills that can be improved concurrently. To determine this, it is important to investigate whether training using a combination of different games can lead to improvements in multiple areas of human cognition.
